# The CONUT Score Independently Predicts Mortality in Older Patients with Hip Fracture

**DOI:** 10.3390/medicina62071275

**Published:** 2026-07-02

**Authors:** Elisa García-Tercero, Alejandro Valcuende-Rosique, Daniela Villalón-Rubio, Ángel Belenguer-Varea, Javier Valcuende-Rosique, Magdalena Linge-Martin, José Viña-Ribes, Francisco José Tarazona-Santabalbina

**Affiliations:** 1 Geriatrics Department, University Hospital of La Ribera, 46600 Alzira, Valencia, Spain; elisagt90@gmail.com (E.G.-T.); valcuende_ale@gva.es (D.V.-R.); belenguer_angvar@gva.es (Á.B.-V.); linge_mag@gva.es (M.L.-M.); fjtarazonas@gmail.com (F.J.T.-S.); 2 Grupo Valenciano de Investigación Clínica en Fragilidad y Síndromes Geriátricos (FRAGIVAL), Centro de Investigación Biomédica en Red de Fragilidad y Envejecimiento Saludable (CIBERFES), 28029 Madrid, Spain; 3 School of Medicine, Universidad Católica de Valencia Sant Vicent Mártir, 46001 Valencia, Spain; 4 Pharmacy Department, Hospital Pare Jofré, 46017 Valencia, Spain; 5 Department of Cardiology, Hospital Clínico Universitario Lozano Blesa, 50009 Zaragoza, Spain; javier.valcuende@gmail.com; 6 Department of Physiology, University of Valencia, 46010 Valencia, Spain; rosique_dol@gva.es

**Keywords:** hip fracture, malnutrition, CONUT score, mortality, older patients

## Abstract

*Background and Objectives:* Malnutrition is highly prevalent among older adults with hip fracture and is associated with poorer surgical outcomes, yet its prognostic relevance is often under-recognized in routine orthopaedic practice. The Controlling Nutritional Status (CONUT) score is an objective laboratory-based screening tool; however, evidence regarding its value for predicting long-term mortality after hip fracture remains limited. This study aimed to evaluate whether nutritional status assessed by the CONUT score independently predicts mortality in older patients with hip fracture. *Materials and Methods:* This retrospective observational cohort study included consecutive patients aged ≥70 years admitted for hip fracture to a tertiary hospital between 2014 and 2021. Nutritional status was assessed at admission using the CONUT score and categorized as no, mild, moderate, or severe nutritional risk. Demographic characteristics, comorbidity burden, perioperative variables, postoperative morbidity, and mortality up to five years were recorded. Survival was evaluated using Kaplan–Meier methods, and independent predictors of mortality were identified using multivariable Cox proportional hazards models adjusted for clinically relevant confounders. *Results:* A total of 2798 patients were included (mean age [SD] 84.3 [6.3] years; 26.4% male), of whom 79.2% presented some degree of nutritional risk at admission. Mortality increased overall with worsening nutritional status (*p* < 0.001). After comprehensive multivariable adjustment, higher CONUT scores remained independently associated with mortality, with each one-point increase associated with an approximately 22% higher risk of long-term death. Poorer nutritional status was also associated with higher postoperative complication rates, greater transfusion requirements, and longer hospital stay. *Conclusions:* Nutritional status assessed using the CONUT score is an independent predictor of short-, mid-, and long-term mortality in older patients undergoing surgery for hip fracture. Incorporation of objective nutritional screening into orthogeriatric pathways may improve perioperative risk stratification and support targeted multidisciplinary management.

## 1. Introduction

Hip fracture represents a major global public health challenge and one of the most severe complications of osteoporosis, with substantial clinical and socioeconomic consequences [1,2]. In Spain, epidemiological estimates from national registry reports published in the late 2010s indicate an incidence of approximately 104 cases per 100,000 inhabitants and an annual healthcare cost of around €1.59 billion, reflecting the growing burden of fragility fractures in an ageing population [3]. Rising life expectancy is expected to further increase the incidence of hip fractures, particularly among individuals aged over 80 years, who face a higher risk of perioperative complications, functional decline, and mortality [4,5]. Indeed, up to one third of patients fail to regain their pre-fracture functional status, and many require prolonged institutional care or long-term support following surgery [2,6].

Multiple factors have been associated with functional outcomes and morbidity and mortality following hip fracture, including cognitive status, demographic characteristics, clinical factors, functional capacity, social determinants, and healthcare-related variables [7,8]. In this context, the identification of modifiable prognostic factors is particularly relevant to prevent a worse evolution of these patients. Among these factors, nutritional status has emerged as a key determinant of clinical outcomes and mortality after hip fracture [6,9,10] as well as after other surgical procedures [11]. Malnutrition, sarcopenia, and frailty represent interconnected dimensions of biological vulnerability that reduce physiological reserve and limit the capacity to withstand surgical stress and postoperative recovery. Previous research has linked malnutrition to dysregulated inflammatory responses, delayed bone healing, and progressive loss of muscle mass and strength, factors that may negatively affect mobility and functional outcomes after hip fracture [12,13].

Despite its well-established impact on morbidity and mortality, malnutrition remains underdiagnosed in the hospital setting. Consequently, the European Society for Clinical Nutrition and Metabolism (ESPEN) [14] guidelines recommend early nutritional screening in patients undergoing surgical procedures using validated tools, in order to identify patients at risk or with established malnutrition and implement appropriate interventions to reverse or mitigate its effects.

Among the validated tools for nutritional assessment, the Controlling Nutritional Status (CONUT) score allows for retrospective and examiner-independent evaluation using routinely available laboratory parameters [15]. It is calculated from serum albumin, total cholesterol concentration, and total lymphocyte count, and classifies patients into four nutritional risk categories: no risk, mild, moderate, and severe. The CONUT score has demonstrated good diagnostic performance for identifying nutritional risk, with reported sensitivity and specificity reaching approximately 80% in different clinical settings. Accordingly, it is primarily considered a first-line screening tool to identify patients who may require a more comprehensive nutritional assessment, and its prognostic value has been demonstrated in various surgical populations [9,11,16].

Although recent studies have explored the association between nutritional status and outcomes after hip fracture, most have focused on short-term endpoints, functional recovery, or smaller cohorts, limiting their ability to define the long-term prognostic value of objective laboratory-based tools such as the CONUT score [10,16]. Consequently, uncertainty remains regarding the independent contribution of nutritional status to long-term mortality within contemporary orthogeriatric care pathways. The present study was therefore designed to evaluate whether baseline CONUT score independently predicts short-, mid-, and long-term mortality in a large cohort of older patients with hip fracture, while also examining its association with postoperative morbidity. We hypothesized that higher CONUT scores at admission would be independently associated with increased mortality risk across all follow-up periods.

## 2. Materials and Methods

### 2.1. Study Design

This retrospective observational cohort study was conducted at Hospital Universitario de la Ribera, a tertiary referral center in Alzira (Valencia, Spain), using prospectively collected data from an institutional orthogeriatric registry embedded in routine clinical practice. The study included consecutive older patients admitted for hip fracture who underwent surgical treatment between January 2014 and December 2021. The hospital follows an established orthogeriatric care model in which patients are jointly managed by traumatologists, geriatricians, nursing staff, and rehabilitation specialists through coordinated and protocolized care pathways.

Upon admission, all patients underwent a comprehensive geriatric assessment performed by a geriatrician within the first 24 h, including evaluation of functional, cognitive, nutritional, and clinical status. Surgical and perioperative management decisions were made through interdisciplinary consensus. Early postoperative mobilization and individualized rehabilitation programs were initiated within the first 48 h after surgery whenever clinically feasible.

### 2.2. Study Participants

Patients aged 70 years or older admitted with a diagnosis of hip fracture between January 2014 and December 2021, treated surgically during the study period, and with available baseline laboratory data required for CONUT score calculation were eligible for inclusion. Patients with fractures secondary to high-energy trauma, polytrauma, pathological fractures, missing baseline laboratory data required for CONUT score calculation, or an estimated life expectancy of less than six months due to any cause were excluded, as these situations reflect end-of-life trajectories or non-comparable clinical scenarios in which mortality is predominantly driven by advanced underlying disease rather than perioperative or nutritional factors.

**Figure 1:**
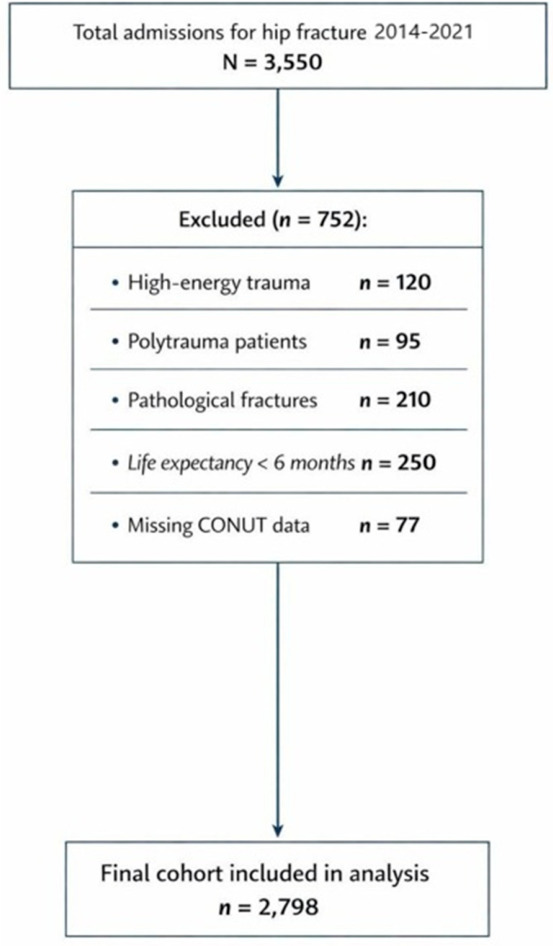
Study flow diagram of patient inclusion and exclusion.

Baseline demographic data, comorbidities, and laboratory parameters were obtained from electronic medical records. Nutritional status at admission was assessed using the Controlling Nutritional Status (CONUT) score, calculated from serum albumin, total cholesterol, and total lymphocyte count. According to established thresholds, patients were categorized as having no nutritional risk, mild, moderate, or severe nutritional risk [15]. Comorbidity burden was assessed using the Charlson Comorbidity Index [17]. The age threshold of 70 years was selected because the institutional orthogeriatric care pathway is systematically implemented from this age onwards, reflecting routine clinical practice at our center and ensuring a homogeneous geriatric population undergoing standardized multidisciplinary management. The patient selection process, including the predefined inclusion and exclusion criteria, is summarized in Figure 1.

### 2.3. Outcomes

The primary outcome was all-cause mortality during follow-up, evaluated at short-term (in-hospital and 30-day), mid-term (90, 180, and 365 days), and long-term (2- and 5-year) intervals according to baseline CONUT nutritional risk.

Secondary outcomes included in-hospital morbidity, comprising overall postoperative complications and major complications, intensive care unit admission, length of hospital stay, red blood cell transfusion requirements, and hospital readmissions. Postoperative complications were classified according to the Clavien–Dindo grading system; major complications were defined as those graded ≥III, reflecting events requiring surgical, endoscopic, or radiological intervention, life-threatening complications, or death. The association between baseline nutritional status and postoperative morbidity was subsequently evaluated.

### 2.4. Data Collection

Data were extracted from electronic medical records and hospital discharge databases by trained investigators using a predefined standardized protocol embedded within the institutional orthogeriatric registry. All study variables were systematically recorded as part of routine clinical care pathways, minimizing interobserver variability during data collection. Variables collected included age, sex, comorbidities, anesthetic risk, laboratory parameters at admission and at hospital discharge, surgical characteristics, postoperative complications, and mortality outcomes. Laboratory values used to calculate the CONUT score were obtained from the hospital’s central laboratory, which operated under standardized analytical procedures and consistent reference ranges throughout the study period; CONUT at admission was calculated using the first available laboratory assessment, whereas CONUT at discharge was derived from the last laboratory evaluation prior to discharge.

Mortality and long-term outcomes were ascertained through linkage with hospital records and regional electronic health databases, enabling systematic verification of vital status throughout follow-up. The use of unique patient identifiers within a universal public healthcare network ensured minimal loss to follow-up and reliable ascertainment of survival outcomes. Patients with missing baseline laboratory data required for CONUT score calculation were excluded prior to analysis. For the remaining study cohort, all variables included in the statistical analyses were complete; therefore, complete-case analyses were performed and no imputation of missing data was required.

### 2.5. Statistical Analysis

Statistical analyses were performed using IBM SPSS Statistics^®^ version 23 (IBM Corp., Armonk, NY, USA). Categorical variables are presented as frequencies and percentages, and continuous variables as means with standard deviations or medians with interquartile ranges according to distribution normality assessed by the Kolmogorov–Smirnov test. Comparisons across CONUT nutritional risk categories were performed using chi-square tests for categorical variables and analysis of variance or Kruskal–Wallis tests for continuous variables, as appropriate.

Survival was evaluated using Kaplan–Meier methods, and differences between groups were assessed with the log-rank test. Patients were followed from hospital admission until death or the end of the study period, and individuals without complete five-year follow-up were administratively censored at the time of their last available survival assessment.

Cox proportional hazards regression models were constructed to evaluate the association between nutritional status and mortality, reporting hazard ratios (HR) with 95% confidence intervals (CI). Multivariable models were adjusted for clinically relevant confounders, including age, sex, Charlson Comorbidity Index, ASA classification, surgical delay, and red blood cell transfusion requirement. These variables were selected based on their established prognostic relevance in older patients with hip fracture and to determine whether the association between the CONUT score and mortality remained independent of major perioperative risk factors. The proportional hazards assumption was verified before model fitting. A backward stepwise selection procedure was applied to derive the final multivariable model, and collinearity among covariates was assessed before model fitting. A two-sided *p*-value < 0.05 was considered statistically significant.

A post hoc power assessment was performed based on the observed mortality rates across CONUT categories. Using two-sample proportion tests, achieved power for 30-day mortality comparisons was 0.86 for no nutritional risk versus mild risk, >0.99 for no risk versus moderate risk, and 0.67 for no risk versus severe risk. For 1-year mortality, achieved power was 0.99, >0.99, and 0.71, respectively. Power estimates derived from the Schoenfeld approximation indicated >0.99 power for all adjusted Cox proportional hazards models, suggesting adequate statistical power for most comparisons except for the severe nutritional risk subgroup.

### 2.6. Ethical Considerations

The study was approved by the Clinical Research Ethics Committee of Hospital Universitario de la Ribera (approval code: HULR23122022) and conducted in accordance with the Declaration of Helsinki and applicable data protection regulations. Given the observational nature of the study, informed consent was waived. Patient confidentiality was ensured through anonymization of all collected data and secure database management.

## 3. Results

A total of 2798 patients aged over 70 years admitted for hip fracture between 2014 and 2021 were included (Figure 1). According to the initial CONUT score, 581 patients (20.8%) had no nutritional risk, while 1700 (60.8%) had mild risk, 455 (16.3%) moderate risk, and 62 (2.2%) severe nutritional risk. Baseline characteristics are presented in Table 1.

The mean (SD) age of the cohort was 84.3 (6.3) years, with a progressive increase in age as nutritional risk worsened (*p* < 0.001). The proportion of male patients was significantly higher in the moderate and severe nutritional risk groups (*p* < 0.001). Comorbidity burden, assessed using the Charlson Comorbidity Index, increased significantly with worsening nutritional status (*p* < 0.001), as did the proportion of patients with high anesthetic risk (ASA ≥ 3).

Among the medical comorbidities analyzed, only chronic renal failure showed a higher prevalence in patients with poorer nutritional status (*p* = 0.003). No significant differences were observed in the prevalence of heart failure, chronic obstructive pulmonary disease, prior stroke, dementia, or diabetes mellitus.

During hospitalization, patients with higher nutritional risk experienced significantly worse clinical outcomes (Table 2). Admission to the intensive care unit increased progressively with CONUT severity, reaching 9.7% in the severe nutritional risk group (*p* < 0.001).

The overall postoperative complication rate was 49.7% and was significantly higher in patients with moderate and severe nutritional risk (*p* < 0.001). Similarly, the proportion of major complications increased progressively from 41.8% in patients without nutritional risk to 72.6% in those with severe nutritional risk (*p* < 0.001). After adjustment for age, sex, Charlson Comorbidity Index, anesthetic risk, and red blood cell transfusion, higher CONUT scores remained independently associated with an increased risk of overall in-hospital complications. Each one-point increase in CONUT was associated with a 6% increase in the odds of complications (adjusted OR 1.06; 95% CI: 1.01–1.11; *p* = 0.011).

Regarding specific complications, poorer nutritional status was associated with a higher incidence of respiratory events (*p* = 0.013), surgical site infections (*p* = 0.016), and cardiovascular complications (*p* = 0.011). The requirement for red blood cell transfusion also increased progressively across nutritional risk categories, reaching 80.6% among patients with severe CONUT scores (*p* < 0.001). Length of hospital stay similarly increased with worsening nutritional status, from a mean (SD) of 7.7 (2.7) days in patients without nutritional risk to 10.1 (6.6) days in those with severe nutritional risk (*p* < 0.001). Readmission rates after hospital discharge were comparable across nutritional risk groups, with no significant differences observed (20.3% overall; *p* = 0.543) (Table 2).

Mortality increased progressively and consistently with worsening baseline nutritional status (Table 3). In-hospital mortality was 1.6% in patients without nutritional risk, compared with 3.5%, 7.7%, and 11.3% in the mild, moderate, and severe risk groups, respectively (*p* < 0.001).

Similarly, 30-, 90-, 180-, and 365-day mortality rates were significantly higher in patients with moderate and severe nutritional risk (all *p* < 0.001). At mid-term follow-up, 2-year mortality reached 54.3% in patients with moderate nutritional risk and 40.3% in those with severe risk, compared with 23.9% in patients without nutritional risk (*p* < 0.001). At 5 years, mortality was 41.8% in the no-risk group and exceeded 60% in patients with moderate and severe nutritional risk (*p* < 0.001).

**Figure 2:**
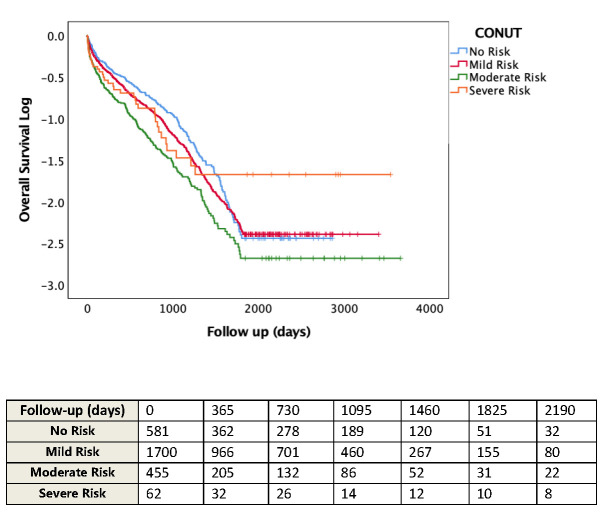
Kaplan–Meier survival log curves according to baseline CONUT nutritional risk.

Survival analysis demonstrated significant differences between groups from the early phases of follow-up (log-rank *p* < 0.001). Median (IQR) overall survival was 677.1 (79.4) days in patients without nutritional risk, decreasing to 267.0 (47.0) days in the moderate-risk group and to 388.5 (187.8) days in the severe nutritional risk group (Figure 2).

Multivariable Cox regression analyses including extended perioperative adjustment (age, sex, Charlson Comorbidity Index, ASA classification, surgical delay, and red blood cell transfusion requirement) confirmed the independent prognostic value of baseline nutritional status. When CONUT was analysed as a continuous variable, each one-point increase was associated with a 21.9% higher risk of long-term mortality (HR 1.22; 95% CI: 1.08–1.37; *p* = 0.001). Increasing age (HR 1.06 per year; 95% CI: 1.03–1.08; *p* < 0.001), higher Charlson Comorbidity Index (HR 1.04; 95% CI: 1.03–1.05; *p* < 0.001), surgical delay (HR 1.04; 95% CI: 1.01–1.07; *p* = 0.006), and red blood cell transfusion requirement (HR 1.30; 95% CI: 1.15–1.47; *p* < 0.001) were also independently associated with mortality, whereas ASA classification did not retain statistical significance after full adjustment.

Consistent results were observed when CONUT was analysed categorically, with mild nutritional risk associated with increased mortality (HR 1.22, 95% CI 1.08–1.38; *p* = 0.001) and moderate nutritional risk showing a stronger association (HR 1.41, 95% CI 1.12–1.76; *p* < 0.001) compared with patients without nutritional risk; in contrast, the severe category did not reach statistical significance after adjustment.

## 4. Discussion

In this retrospective observational study, we demonstrate that nutritional status assessed using the CONUT score is an independent and clinically relevant predictor of short-, medium-, and long-term mortality in patients aged over 70 years admitted with hip fracture. Our findings indicate that even mild nutritional impairment is associated with an increased risk of mortality, whereas moderate and severe malnutrition substantially amplify this risk, after comprehensive multivariable adjustment, independently of age, sex, and comorbidity burden. Notably, five-year mortality was high across all nutritional categories, reflecting the advanced age and clinical complexity of orthogeriatric populations, in whom long-term outcomes are influenced by multiple competing risks and non-nutritional determinants such as frailty and chronic comorbidity. These results reinforce the role of nutritional status as a relevant, yet non-isolated, prognostic component within comprehensive geriatric assessment.

The association between malnutrition and adverse outcomes after hip fracture has been previously described, although most studies have relied on subjective scales or short-term endpoints. Our findings extend this evidence by showing that an objective and easily reproducible laboratory-based tool such as the CONUT score enables stratification of mortality risk over prolonged follow-up. Previous reports by Han et al. [6] and Franz et al. [18] identified malnutrition as a predictor of early mortality but were limited by smaller cohorts and shorter longitudinal observation.

The risk gradient observed according to the degree of nutritional deterioration further supports the biological plausibility of our findings. Importantly, overall mortality increased with worsening nutritional status, yet a strictly monotonic gradient was not observed across all follow-up periods, with mortality estimates in the moderate-risk group occasionally exceeding those of the severe-risk subgroup. This pattern likely reflects the limited number of patients in the severe nutritional risk group (n = 62), resulting in reduced statistical power and greater imprecision of subgroup-specific estimates. Consequently, comparisons involving this subgroup should be interpreted with caution. Malnutrition has been linked in previous research to systemic inflammation, immune dysfunction, accelerated loss of muscle mass, and delayed bone healing—mechanisms that may contribute to poorer recovery after surgical stress. Moreover, the frequent coexistence of malnutrition, sarcopenia, and frailty in older adults generates a state of heightened vulnerability that may explain the progressive increase in postoperative complications and mortality observed in our cohort [4,6,8,19].

These considerations also highlight that the CONUT score may capture biological processes extending beyond nutritional status alone. Although originally developed as a nutritional screening tool, its individual components—serum albumin, total lymphocyte count, and total cholesterol—are also influenced by systemic inflammation, acute illness, and overall physiological reserve [20]. Consequently, the prognostic value of CONUT observed in our study is likely to reflect the combined contribution of nutritional status and these interrelated biological processes, rather than nutrition in isolation.

Postoperative morbidity increased progressively with worsening nutritional status, with higher rates of respiratory, cardiovascular, and infectious complications, as well as greater transfusion requirements and longer hospital stay. These findings are consistent with previous reports and recent systematic reviews linking malnutrition to poorer in-hospital outcomes in the older surgical population [6,21,22]. Importantly, the prognostic contribution of the CONUT score persisted even after extended adjustment for perioperative factors such as anesthetic risk, surgical delay, and transfusion requirement, with each one-point increase associated with an approximately 22% higher risk of long-term mortality. Together, these findings suggest that higher CONUT scores reflect a broader dimension of biological vulnerability not fully captured by traditional surgical risk markers or comorbidity indices.

These observations are in line with previous research in surgical and oncological settings, where the CONUT score has shown good discriminatory capacity for predicting complications and mortality [23,24,25,26]. Within the orthogeriatric field, studies by Cheng et al. [16] and Kotera et al. [27] have also supported its clinical usefulness, albeit with shorter follow-up periods. Compared with other nutritional assessment tools, such as the Mini Nutritional Assessment (MNA) [28] and the Global Leadership Initiative on Malnutrition (GLIM) criteria [29], which require clinical, functional, and anthropometric evaluation, the CONUT score relies exclusively on routinely available laboratory parameters, enabling rapid, objective, and reproducible nutritional screening. Although these tools should be regarded as complementary rather than interchangeable, the simplicity and feasibility of CONUT make it particularly suitable for integration into orthogeriatric care pathways and perioperative risk stratification.

These characteristics have important clinical implications. The high prevalence of malnutrition observed in our cohort—nearly 80% of patients exhibited some degree of nutritional risk—highlights the need to consider nutritional screening as an essential component of comprehensive hip fracture management [4,5,8,30]. The objective and readily available nature of the CONUT score facilitates its implementation in routine clinical practice and enables repeated assessment throughout hospitalization.

The ability to identify high-risk patients at the time of admission opens the door to early and individualized nutritional interventions. Although our study did not specifically evaluate the impact of such interventions, growing evidence suggests that nutritional optimization may improve functional recovery and reduce mortality in hospitalized older adults [31,32]. In this context, reassessment of nutritional status at discharge may represent an additional opportunity to intervene and improve long-term outcomes.

Among the main strengths of this study are the large sample size, the inclusion of consecutive patients managed within a standardized orthogeriatric pathway, systematic nutritional assessment using an objective and reproducible tool, and prolonged longitudinal follow-up.

### Limitations

Several limitations should be acknowledged. First, the single-center design may limit the generalizability of our findings to other healthcare settings. Second, although the overall sample size was large, only 62 patients were classified as having severe nutritional risk. This limited subgroup size reduced statistical power and the precision of subgroup-specific estimates and may explain the inconsistent mortality gradient observed between the moderate- and severe-risk groups at certain follow-up time points. Third, although the multivariable models were adjusted for several clinically relevant covariates, residual confounding cannot be excluded, as important prognostic factors such as frailty, cognitive status, pre-fracture functional capacity, residential status, and sarcopenia were not available for adjustment. Fourth, detailed information regarding nutritional interventions during hospitalization was not available, precluding assessment of their potential influence on clinical outcomes. Finally, the CONUT score was not directly compared with other widely used nutritional assessment tools such as the Mini Nutritional Assessment (MNA) [26] or the Global Leadership Initiative on Malnutrition (GLIM) criteria [27]. Nevertheless, the large cohort, standardized patient management, and prolonged follow-up support the robustness and clinical relevance of our findings.

## 5. Conclusions

Our findings demonstrate that nutritional status assessed using the CONUT score is a powerful independent predictor of mortality and complications in patients aged over 70 years with hip fracture. Systematic incorporation of nutritional screening using objective tools such as CONUT may improve risk stratification and facilitate targeted interventions in this particularly vulnerable population. Future prospective multicenter studies should validate these findings across different healthcare settings and evaluate whether CONUT-guided nutritional interventions can improve not only survival but also functional recovery, postoperative complications, and quality of life.

## Figures and Tables

**Table 1:** Baseline demographic and clinical characteristics according to CONUT nutritional risk categories.

Variable	No Nutritional Risk (n = 581)	Mild Risk (n = 1700)	Moderate Risk (n = 455)	Severe Risk (n = 62)	Global CONUT (n = 2798)	*p*-Value
Age (years), m (SD)	83.5 (6.2)	84.4 (6.3)	85.4 (6.4)	84.7 (6.0)	84.3 (6.3)	<0.001
Male sex, n (%)	95 (16.3%)	438 (25.8%)	184 (40.4%)	22 (35.5%)	739 (26.4%)	<0.001
CCI score, m (SD)	2.3 (2.1)	2.5 (2.3)	3.1 (2.6)	3.3 (3.3)	2.6 (2.4)	<0.001
ASA ≥ 3, n (%)	23 (4.0%)	71 (4.2%)	39 (8.6%)	6 (9.7%)	140 (5.0%)	<0.001
Hx of IHD, n (%)	38 (6.5%)	131 (7.7%)	61 (13.4%)	6 (9.7%)	232 (8.3%)	0.001
Hx of HF, n (%)	36 (6.2%)	111 (6.5%)	27 (5.9%)	7 (11.3%)	179 (6.4%)	0.470
Hx of COPD, n (%)	60 (10.3%)	197 (11.6%)	59 (13.0%)	7 (11.3%)	322 (11.5%)	0.605
Hx of CVA, n (%)	13 (2.2%)	48 (2.8%)	13 (2.9%)	2 (3.2%)	76 (2.7%)	0.840
Hx of Dementia, n (%)	79 (13.6%)	270 (15.9%)	81 (17.8%)	6 (9.7%)	434 (15.5%)	0.154
Hx of RF, n (%)	61 (10.5%)	160 (9.4%)	69 (15.2%)	10 (16.1%)	297 (10.6%)	0.003
Hx of Diabetes, n (%)	141 (24.3%)	490 (28.8%)	144 (31.7%)	17 (27.4%)	789 (28.2%)	0.061

ASA: American Society of Anesthesiologists. CCI: Charlson Comorbidity Index. CONUT: Controlling Nutritional Status. Hx of IHD: History of Ischemic Heart Disease. Hx of HF: History of Heart Failure. Hx of COPD: History of Chronic Obstructive Pulmonary Disease. Hx of CVA: History of Cerebrovascular Accident. Hx of RF: History of Renal Failure. m = mean; SD: Standard Deviation.

**Table 2:** In-hospital outcomes and postoperative complications according to CONUT nutritional risk categories.

	No Nutritional Risk (n = 581)	Mild Risk (n = 1700)	Moderate Risk (n = 455)	Severe Risk (n = 62)	Global CONUT (n = 2798)	*p*-Value
ICU, n (%)	3 (0.5%)	19 (1.1%)	10 (2.2%)	6 (9.7%)	39 (1.4%)	<0.001
CONUT at hospital discharge, n (%)	332 (20.5%)	830 (51.3%)	401 (24.8%)	54 (3.4%)	1617 (100%)	<0.001
Complications, n (%)	249 (42.9%)	821 (48.3%)	275 (60.4%)	45 (72.6%)	1391 (49.7%)	<0.001
Major complications, n (%)	243 (41.8%)	789 (46.4%)	268 (58.9%)	45 (72.6%)	1343 (48.0%)	<0.001
AE Delirium, n (%)	58 (10.0%)	170 (10.0%)	63 (13.9%)	9 (14.5%)	299 (10.7%)	0.111
AE Cardiac, n (%)	24 (4.1%)	104 (6.1%)	41 (9.0%)	6 (9.7%)	173 (6.2%)	0.011
AE Anemia, n (%)	73 (12.6%)	160 (9.4%)	52 (11.4%)	9 (14.5%)	294 (10.5%)	0.089
AE UTI, n (%)	26 (4.5%)	66 (3.9%)	20 (4.4%)	1 (1.6%)	112 (4.0%)	0.695
AE Digestive Issues, n (%)	1 (0.2%)	9 (0.5%)	3 (0.7%)	2 (3.2%)	14 (0.5%)	0.012
AE Respiratory, n (%)	19 (3.3%)	85 (5.0%)	29 (6.4%)	7 (11.3%)	138 (4.9%)	0.013
AE Surgical site infection, n (%)	1 (0.2%)	9 (0.5%)	2 (0.4%)	2 (3.2%)	14 (0.5%)	0.016
Surgery Delay in hours, m (SD)	44.2 (26.9)	45.6 (1.8)	47.3 (32.3)	48.2 (54.9)	45.6 (31.6)	0.422
Operating room time (min), m (SD)	93.1 (25.1)	93.3 (35.0)	90.8 (22.9)	111.2 (45.4)	93.2 (31.8)	<0.001
Surgical time (min), m (SD)	65.2 (24.3)	64.4 (27.5)	60.3 (20.7)	79.7 (38.0)	64.2 (26.3)	<0.001
Surgery Delay exceeding 48 Hours, n (%)	207 (35.6%)	643 (37.8%)	171 (37.6%)	22 (35.5%)	1043 (37.3%)	0.802
Surgery Delay of 72 h, n (%)	86 (14.8%)	269 (15.8%)	82 (18.0%)	11 (17.7%)	448 (16.0%)	0.535
Red blood cell transfusions, n (%)	308 (53.0%)	983 (57.8%)	311 (68.4%)	50 (80.7%)	1651 (59.0%)	<0.001
Initial Hb, m (SD)	12.8 (1.5)	12.4 (1.6)	11.8 (1.9)	11.2 (1.9)	12.4 (1.7)	<0.001
Final Hb, m (SD)	10.4 (1.1)	10.4 (1.2)	10.2 (1.1)	10.1 (1.1)	10.3 (1.2)	0.006
Initial Glomerular Filtration (mil/min), m (SD)	63.4 (23.4)	65.1 (24.3)	62.2 (26.5)	63.5 (32.0)	64.3 (24.7)	0.102
Final Glomerular Filtration (mil/min), m (SD)	82.5 (36.6)	81.1 (37.1)	77.3 (39.3)	80.6 (47.6)	80.0 (37.6)	0.162
Chronic kidney disease stage ≥ 3, n (%)	269 (46.3%)	734 (43.2%)	222 (48.8%)	34 (54.8%)	1260 (45.0%)	0.018
Readmissions after Hospital Discharge, n (%)	115 (19.8%)	338 (19.9%)	104 (22.9%)	12 (19.4%)	569 (20.3%)	0.543
Reoperations, n (%)	9 (1.5%)	20 (1.2%)	9 (2.0%)	3 (4.8%)	41 (1.5%)	0.081
Total length of stay, m (SD)	7.7 (2.7)	8.0 (4.3)	8.6 (4.3)	10.1 (6.6)	8.1 (4.1)	<0.001
Number of diagnoses, m (SD)	10.8 (4.6)	11.7 (5.0)	12.4 (5.2)	12.8 (6.1)	11.6 (5.0)	<0.001

AE: adverse event. Hb: Hemoglobin. ICU: intensive care unit. m = mean. min: minutes. SD: Standard Deviation. UTI: Urinary Tract Infection.

**Table 3:** Mortality outcomes stratified by CONUT nutritional status.

	No Nutritional Risk (n = 581)	Mild Risk (n = 1700)	Moderate Risk (n = 455)	Severe Risk (n = 62)	Global CONUT (n = 2798)	*p*-Value
In-hospital mortality, n (%)	9 (1.6%)	59 (3.5%)	35 (7.7%)	7 (11.3%)	109 (3.9%)	<0.001
30-Day Mortality, n (%)	21 (3.6%)	112 (6.6%)	72 (15.8%)	9 (14.5%)	214 (7.7%)	<0.001
90-Day Mortality, n (%)	47 (8.1%)	229 (13.5%)	114 (25.1%)	14 (22.6%)	404 (14.4%)	<0.001
180-Day Mortality, n (%)	71 (12.2%)	315 (18.5%)	155 (34.1%)	16 (25.8%)	557 (19.9%)	<0.001
365-Day Mortality, n (%)	98 (16.9%)	425 (25.0%)	198 (43.5%)	20 (32.3%)	741 (26.5%)	<0.001
2-Year Mortality, n (%)	139 (23.9%)	581 (34.2%)	247 (54.3%)	25 (40.3%)	992 (35.5%)	<0.001
5-Year Mortality, n (%)	243 (41.8%)	903 (53.1%)	320 (70.3%)	38 (61.3%)	1504 (53.8%)	<0.001

## Data Availability

Data supporting the findings of this study are available from the corresponding author upon reasonable request.

## Abbreviations

AE	Adverse Event
ASA	American Society of Anesthesiologists
CCI	Charlson Comorbidity Index
CI	Confidence Interval
CONUT	Controlling Nutritional Status
COPD	Chronic Obstructive Pulmonary Disease
CVA	Cerebrovascular Accident
GLIM	Global Leadership Initiative on Malnutrition
Hb	Hemoglobin
HF	Heart Failure
HR	Hazard Ratio
ICU	Intensive Care Unit
IHD	Ischemic Heart Disease
IQR	Interquartile Range
MNA	Mini Nutritional Assessment
OR	Odds Ratio
RF	Renal Failure
SD	Standard Deviation
UTI	Urinary Tract Infection
